# Testing the Effects of an Introduced Palm on a Riparian Invertebrate Community in Southern California

**DOI:** 10.1371/journal.pone.0042460

**Published:** 2012-08-03

**Authors:** Theresa Sinicrope Talley, Kim-Chi Nguyen, Anthony Nguyen

**Affiliations:** 1 Scripps Institution of Oceanography, University of California San Diego, La Jolla, California, United States of America; 2 Department of Biology, University of San Diego, San Diego, California, United States of America; National Institute of Water & Atmospheric Research, New Zealand

## Abstract

Despite the iconic association of palms with semi-arid regions, most are introduced and can invade natural areas. Along the San Diego River (San Diego, California, USA), the introduced Canary Island date palm (*Phoenix canariensis*) forms dense patches among native riparian shrubs like arroyo willow (*Salix lasiolepis*). The structural differences between the palm and native shrubs are visually obvious, but little is known about palm’s effects on the ecosystem. We tested for the effects of the palm on a riparian invertebrate community in June 2011 by comparing the faunal and environmental variables associated with palm and willow canopies, trunks and ground beneath each species. The palm invertebrate community had lower abundance and diversity, fewer taxa feeding on the host (e.g., specialized hemipterans), and more taxa likely using only the plant’s physical structure (e.g., web-builders, oak moths, willow hemipterans). There were no observed effects on the ground-dwelling fauna. Faunal differences were due to the physical and trophic changes associated with palm presence, namely increased canopy density, unpalatable leaves, trunk rugosity, and litter accumulations. Palm presence and resulting community shifts may have further ecosystem-level effects through alteration of physical properties, food, and structural resources. These results were consistent with a recent study of invasive palm effects on desert spring arthropods, illustrating that effects may be relatively generalizable. Since spread of the palm is largely localized, but effects are dramatic where it does occur, we recommend combining our results with several further investigations in order to prioritize management decisions.

## Introduction

Images of many semi-arid and/or semi-tropical regions would not be complete without picturesque palms, but in areas such as California these palms are mostly non-native. Tropical icons, such as the fan palms (*Washingtonia* spp.*)* and the date palms (*Phoenix* spp.*)*, are commonly used in landscaping [1] and are making their way throughout relatively moist natural areas [2], such as desert springs in California [3] and riparian corridors of California and Florida [4]–[5]. In California desert springs, two introduced palms (*W. filifera* and *P. dactylifera* L.) outcompete native vegetation for sunlight, space, and water; and thick layers of wide, fibrous palm frond litter prevent new plants from growing [3], [6]–[7]. These desert invaders were also associated with lower abundances and diversity of arthropods, and fewer herbivores than on native plant species [3]. The effects of palm invasions in riparian corridors, however, are uncertain despite the higher prevalence of these ecosystems and their vulnerability to invasion [8].

The Canary Island date palm (*P. canariensis*) is a particularly popular landscaping species in warm climates throughout the world [1], especially California [1], [9]–[10], making it a common invader of moist ecosystems within these regions, such as along the San Diego River. Unassisted large-scale spread is relatively slow, especially compared to more aggressive invaders (e.g., the giant reed grass, *Arundo donax*), but localized spread and dense patch formation are common along Southern California rivers due to the high propagule pressure of horticultural sources, the high fecundity of established adults [2], [11]–[12] and moderate salt tolerance [13]–[14] allowing it access to estuarine river reaches [15].

**Figure 1:**
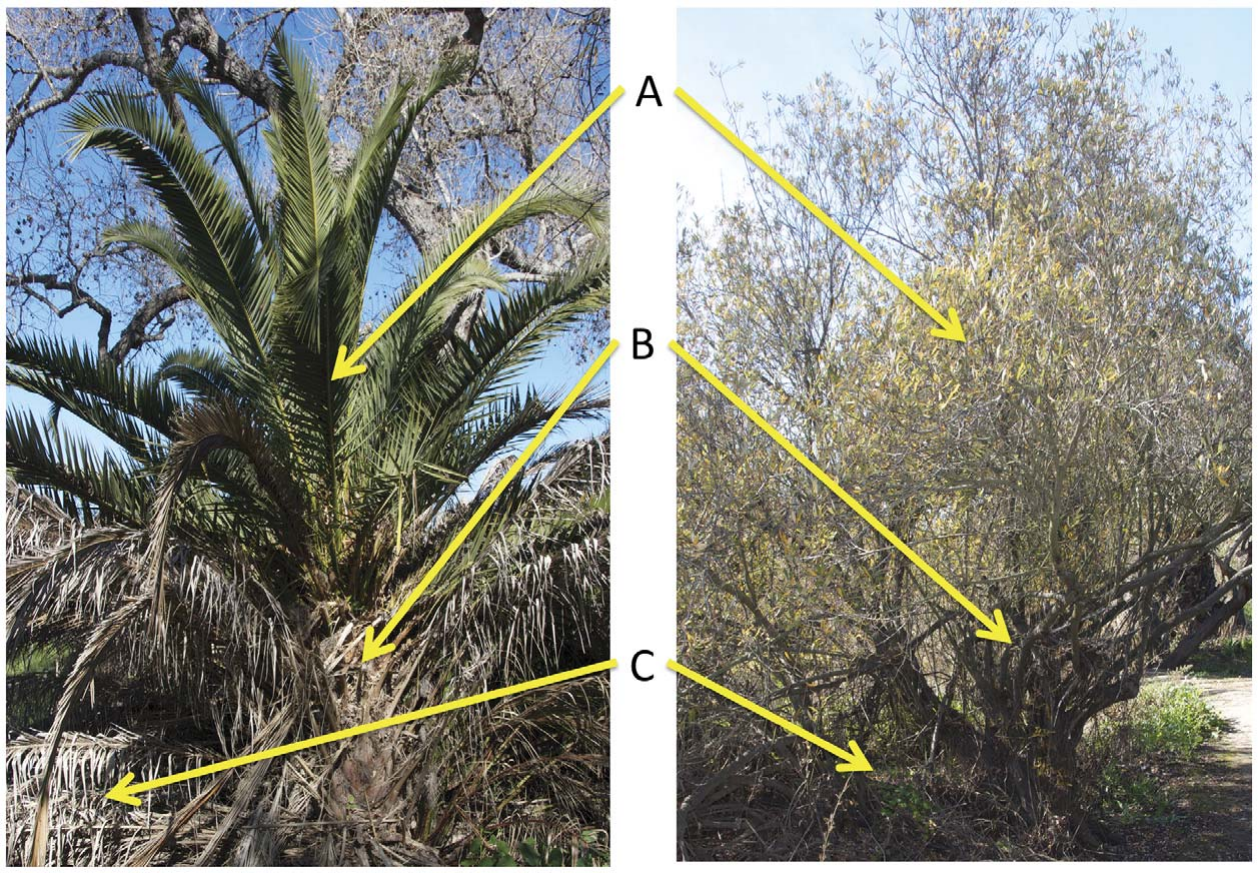
Structural differences between an introduced palm and native willow. Photographs highlighting the structural differences in the canopies (A), trunks (B), and litter (C) of the introduced Canary Island date palm (left) and the native arroyo willow (right) along the San Diego River, California, USA. The front of the palm trunk had been trimmed revealing the branching structure – see side of plant for denseness of canopy. Note ground under willow is patchy with plants and thin patches of leaf litter while thick litter accumulations occur under palm with no understory growth. Photos are from July 2011.

On the San Diego River floodplain, the Canary Island date palm (hereafter “the palm”) is found at mid-riparian elevations and often in association with arroyo willow (*Salix lasiolepis*) [15]. In areas where palm establishment has occurred, the structural changes due to differences in the canopy, trunk and litter are visible at a glance (Fig. 1) and likely influence associated fauna by changes to primary production food sources and/or living space (e.g., [3], [16]–[18]). The long, tough, and dense fronds of the palm canopy contrast with the airy, complex networks of willow branches and small soft leaves (Fig. 1A), a similar contrast to that observed between palms and native desert spring shrubs [3]. The palm trunk is extremely rugous with frond bases that capture detritus, while that of the willow is relatively smooth and branching (Fig. 1B). The ground beneath the palm is carpeted with the tough, fallen fronds while there is a thinner layer of small-leaf litter in various states of decomposition lies beneath the willow (Fig. 1C). Palm presence, therefore, potentially alters the type, amount, and quality of available primary production, including increasing relative amounts of tough, little-digestible leaves and leaf litter, decreasing herbaceous understory cover, and increasing detritus trapped on the rugous trunk (e.g., [3], [19]–[20]). Living space (physical structure) is also likely altered with palm presence due to the seemingly denser, larger, simpler (less branching) canopy, more trunk surface rugosity, and greater accumulations of litter and debris (e.g., [21]–[22]).

### Goals and Objectives

The main goal of this study was to test the effects of the invasion of Canary Island palm on a riparian invertebrate community. Invertebrates often closely track environmental changes and can reveal the ways in which changes to physical properties and basal resources, such as those that occur with plant invasions, may affect the greater ecological community [23]. Our goal was met by testing for differences (or similarities) in invertebrate community variables (abundance, diversity, composition) associated with the canopy, trunk, and ground beneath the canary island palm and the native arroyo willow.

## Materials and Methods

This study was conducted during June 2011 in a 9.7 ha area of riparian woodland within the Mission Valley Preserve, a public park located on the lower San Diego River (32°45′44.56″N, 117°11′37.72″W). Permission to perform studies in this area was granted by the City of San Diego, owners and managers of the land, and the San Diego River Park Foundation, managers of the park. No other special permits were required, and no protected species were involved. Fifteen pairs of Canary Island palm and nearby (10–20 m apart) arroyo willow shrubs were selected. Pairs were at least 50 m apart. The canopy, stems and ground beneath the dripline of each woody plant were sampled for invertebrate community and environmental variables.

### Invertebrate Community

#### Canopy and trunk dwelling fauna

Each plant canopy and trunk was sampled mid-day (10 am – 2 pm) over two consecutive sunny, warm (24–26 deg C) days using a leaf blower that had been modified to vacuum [24]. Each canopy and trunk was sampled from the ground by continuously passing the vacuum over green foliage (canopy) or live main and secondary stems (trunk) for 30 seconds. When possible, invertebrates were identified, enumerated and released in the field. Remaining samples were transferred to zipped plastic bags and kept cool until frozen in the lab. Invertebrates were sorted from plant material and debris, enumerated and identified to the lowest taxonomic group possible using a stereomicroscope.

#### Ground-dwelling fauna

Ground-dwelling fauna were sampled using pitfall traps, each consisting of a 15 cm-diameter ×15 cm-deep bucket with a lid suspended 2 cm above the opening to reduce escape of flying invertebrates and predation by larger animals, and green leaves placed in the bottom as a refuge to reduce risks of desiccation and within-trap predation. One pitfall trap per plant was set in the ground beneath the dripline and left in the field for 6 days. When possible, invertebrates were identified, enumerated and released in the field. The remaining samples were transferred to zipped plastic bags and kept cool until frozen in the lab. Invertebrates were sorted from plant material and debris, enumerated and identified to the lowest taxonomic group possible and/or separated by the morpho-species concept using a stereomicroscope.

### Environmental Variables

The differences in structure and resulting physical and biological properties between each woody species was quantified by measuring the physical properties of each plant, the biomass of plant litter, and the percent ground cover and light attenuation under each canopy.

Plant physical properties taken per plant consisted of height, measured using a clinometer, canopy area (longest diameter × perpendicular diameter) measured with measuring tape, and trunk diameter at ground level, measured with a diameter tape. Five subsamples of leaf toughness, measured with a penetrometer, and trunk rugosity were taken per plant and averaged. Trunk rugosity was estimated using a 100 cm long string, run vertically and in contact with the surface of each trunk at about chest height. The distance between each end of the string was then measured and subtracted from the 100 cm total length to give an estimate of amount of rugosity (e.g., length of string run over bumps). Rugosity index is presented as a percent (length of string run over bumps/total length of string).

The biomass of plant litter for each plant was estimated by clipping (if needed) and collecting all litter within three 0.25 m^2^ quadrat subsamples, weighing the biomass in the field with a handheld scale, and averaging the three subsamples. Percent ground cover under the dripline of each plant was estimated by averaging visual estimates of cover within three 0.25 m^2^ quadrats (% open space, % annual grasses, % litter). Photosynthetic-light attenuation of each plant canopy was measured using an Apogee Quantum handheld light meter during mid-day (10 am –2 pm) over two consecutive sunny days. Three measures were taken at each plant, standardized to full sunlight (under-canopy light [µmol photons m^−2^ s^−1^]/full sunlight [µmol photons m^−2^ s^−1^]), and averaged.

### Statistics

All data were log (x+1) transformed or arcsin square root transformed (proportion data) before analyses [25]. Differences in environmental variables, and invertebrate total abundance, diversity (no. species per sample and H’), and proportions of major taxonomic groups (usually Order) between palm and arroyo willow were performed using paired t-tests in JMP® 9 Statistical Software.

Multivariate analyses were carried out on invertebrate assemblages of the palm and willow plants using Primer Software [26] and species lists with counts (no abundance cut-off, standardization or weighting was used). A total of 83 canopy species, 63 trunk species and 24 ground-dwelling species were found and used for all of the multivariate analyses. Bray-Curtis similarity indices of log (x+1) transformed, unstandardized, unrelativized data were calculated to compare communities and relate them to environmental variables. There were 5.7% zeros and a coefficient of variation (CV) of 52% for the canopy faunal matrix, 12% zeros and CV = 76% for the trunk faunal matrix and 38% zeros and CV = 99% for the ground faunal matrix. Community similarities and differences were explored by non-metric multidimensional scaling (MDS; see [27]) on the Bray-Curtis similarity indices. Six different random staring points with up to 1,000 steps were used. The stress values from the six runs were examined for stability to determine whether a global solution had been found. Only analyses with stress values of <0.2 were used. Stress is a measure of how well the solution (in this case the two-dimensional MDS plots) represents the distances between the data (roughly, the percentage of variance not explained by all dimensions in the MDS.) [27] suggests values <0.1 are good and <0.2 are useful.

Significance testing for differences in faunal composition between palm and willow plants and between pair number, or site (i.e., location), were done using an analysis of similarity (ANOSIM) procedure. This is a randomized permutation test based on rank similarities of samples [27]. The significance level of resultant pairwise comparisons were adjusted by Bonferroni correction for the number of comparisons made. Analyses of invertebrate dissimilarities between palm and willow (faunal composition among pair numbers was not significant), and the particular taxa contributing to the dissimilarity, were carried out using SIMPER on the Bray-Curtis matrices [27]. The SIMPER results specify which taxa are responsible for the ANOSIM results by comparing the average abundances of taxa between palm and willow assemblages. The average dissimilarity between samples from the two groups (palm and willow) is computed and then broken down into contributions from each species. Those species with high average terms relative to the standard deviation are important in the differentiation of willow and palm assemblages.

Relationships between the environmental variables and species diversity and total abundance were tested with forward, stepwise multiple regressions using JMP® 9 Statistical Software. Variables included in the model met the criteria of p≤0.05 and r^2^≥0.04. Tests of the environmental variables that best explain community differences were conducted using the Bray-Curtis similarity indices with the BEST Analysis in Primer Software using the BVSTEP method (criteria: rho >0.95, delta rho <0.001) with fixed starting variables and a Euclidean distance resemblance measure. BVSTEP sequentially adds environmental variables, keeping those that best explain faunal community patterns and eliminating those that explain least. Several iterations of the test are run with a random selection of variables to ensure that the best match is found [26], [27]. The environmental variables tested are listed in Table 1.

**Table 1:** Differences as revealed by paired t-test analyses in physical and biological environmental variables associated with the introduced Canary Island date palm and native arroyo willow found along the lower San Diego River, California in June 2011.

	Willow		Palm	Paired t-test statistics			
variable tested	mean ± 1SE		mean ± 1SE	P	t	df	n
*Canopy properties*							
height		4.2±0.3 m	4.7±0.3 m	0.130	1.6	14	15
canopy area		50.0±6 m^2^	61.6±3 m^2^	0.046	2.2	14	15
light attenuation		93±0.4%	97±0.3%	<0.001	10.2	14	15
leaf toughness		0.12±0.01 kg cm^−2^	2.0±0.2 kg cm^–2^	<0.001	9.9	14	15
*Trunk properties*							
trunk diameter		41±4 cm	134±14 cm	<0.001	6.2	14	15
trunk rugosity		4±0.7%	38±1%	<0.001	17.6	14	15
*Understory properties*							
litter biomass		178±30 g 0.25 m^−2^	340±39 g 0.25 m^−2^	<0.001	4.4	14	15
% grass cover		41±9%	16±7%	0.018	2.7	14	15
% litter cover		41±9%	61±8%	0.084	1.9	14	15
% pen space		18±4%	23±6%	0.450	0.8	14	15

## Results

### Physical and Biological Environments

The palm had on average a 24% larger canopy area and 3 times wider trunk than the willow (Table 1). The greater aboveground stature and dense fronds of the palm compared with willow resulted in 7% less light penetration through the canopy, 20% more litter accumulation, and 25% less grass cover (Table 1). Palm trunks were also nearly 10 times as rugous (amount of non-branch structure on stems) as those of the willow (Table 1).

### Invertebrate Abundance and Diversity

The palm had only about 60% of the invertebrate abundance and 60–70% of the species per sample than the willow (Table 2). Diversity including evenness (H’) of the palm invertebrate assemblages was 70–80% of those in the willow, although this value (H’) was low for the ground-dwelling faunal community of both plants (Table 2).

**Table 2:** Differences as revealed by paired t-test analyses in invertebrate abundance and diversity associated with A.) canopies, B.) trunks, and C.) ground beneath the introduced Canary Island date palm and native arroyo willow along the lower San Diego River, California, USA in June 2011.

A. Canopy						
	Willow	palm	Paired t-test statistics			
	mean ± 1SE	mean ± 1SE	P	t	df	n
total abundance	44.4±6.2	28.9±5.7	0.045	2.2	14	15
no. species	13.0±1.1	8.8±0.7	0.010	3.0	14	15
H’	2.1±0.08	1.7±0.10	0.046	2.2	14	15
**B. Trunk**						
	**Willow**	**palm**	**Paired t-test statistics**			
	**mean ±** **1SE**	**mean ±** **1SE**	**P**	**t**	**df**	**n**
total abundance	19.9±2.8	11.5±1.7	0.038	2.3	14	15
no. species	8.7±0.7	5.4±0.7	0.015	2.8	14	15
H’	1.8±0.1	1.3±0.1	0.040	2.2	14	15
**C. Ground**						
	**Willow**	**palm**	**Paired t-test statistics**			
	**mean ± 1SE**	**mean ± 1SE**	**P**	**t**	**df**	**n**
total abundance	50.3±17.4	33.3±14.1	0.36	0.94	14	15
no. species	3.8±0.3	2.8±0.2	0.02	2.51	13	14
H’	0.8±0.1	0.6±0.1	0.49	0.71	13	14

total abundance: average number of individuals per sample.

no. species, H’: average number of species/H’ per sample.

### Faunal Community Composition

#### Major taxonomic groupings

The palm canopies and/or trunks had higher proportions of arachnids, lepidopterans, neuropterans and psocopterans than willow, while willow canopies and trunks had higher proportions of coleopterans and hymenopterans (Table 3). None of the ground-dwelling taxa differed between palm and willow, with arachnids, hymenoptera (mostly the Argentine ant) and coleopteran dominating the ground beneath both plants (Table 3).

**Table 3:** Average (± 1SE) proportions (%) of major invertebrate taxonomic groups (insect order or chelicerata class) found in the canopy, trunk and ground beneath the Canary Island date palm and the native arroyo willow along the San Diego River, California, USA in June 2011.

	Canopy		Trunk		Ground	
taxonomic group	Willow	palm	willow	palm	willow	palm
Arachnida	3±1	13±4**	5±2	15±4**	33±10	54±12
Coleoptera	5±1	4±1	4±2*	0	9±5	3±2
Collembola	0	2±1	0	0	0	0
Dermaptera	0	0	0	0	<1	0
Diplopoda	0	0	0	<1	<1	<1
Diptera	6±2	9±2	26±6	14±3	<1	0
Hemiptera	29±5	28±7	19±5	22±7	1±1	0
Hymenoptera	28±5***	9±2	30±6**	13±4	51±12	42±12
Lepidoptera	2±1	2±1	6±2	24±7**	1±1	<1
Isopoda	0	0	0	0	3±3	<1
Neuroptera	<1	4±1**	0	0	0	0
Odonata	0	<1	0	0	0	0
Orthoptera	0	0	<1	0	<1	1±1
Psocoptera	6±4	27±7***	4±2	8±4	0	0
Thysanoptera	0	1±1	1±1	<1	0	0
Trichoptera	<1	<1	4±2	1±1	0	0

Differences in the proportion of each taxon between palm and willow as revealed by paired t-test analyses are indicated. Paired t-test statistics: *0.05≤p≤0.10, t = 1.8–2, df = 14, n = 15

** 0.01≤p≤0.05, t = 2.1–3, df = 14, n = 15

*** p<0.01, t = 3.1–7, df = 14, n = 15

#### Species compositions

**Figure 2:**
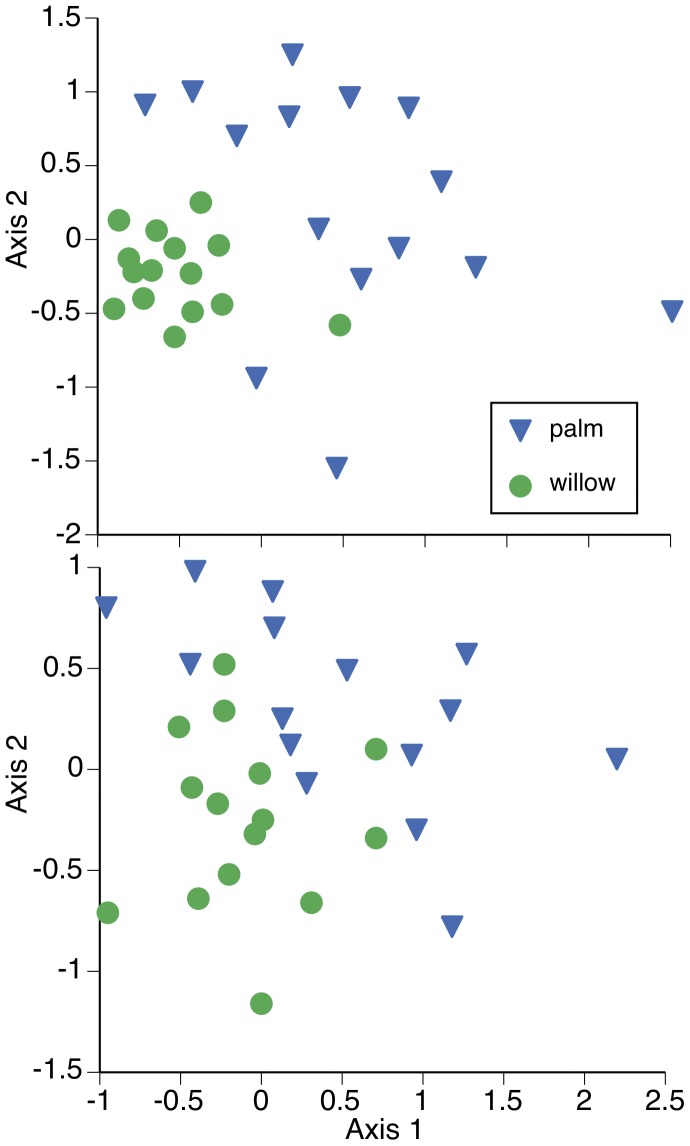
Differences in the invertebrate communities associated with an introduced palm and native willow. Multidimensional scaling plots of the invertebrate communities associated with the canopies (A.) and trunks (B.) of the introduced Canary Island date palm and native arroyo willow along the San Diego River, California, USA. Data are species lists with counts from June 2011. Stress values, or the overall measure of quality of fit of the MDS to the data, are stress = 0.17 for the canopy and stress = 0.19 for the trunk graph. Canopy community differences were correlated with the tougher leaves and rougher trunk of palm than willow, and trunk community differences were correlated with the rougher trunks of palm.

Many of the differences between the faunal assemblages associated with palm and willow occurred at lower taxonomic levels (morpho-species groups), although there was still no difference in the ground-dwelling invertebrate communities beneath the palm and willow (ANOSIM Global P = 0.58). The palm and willow faunal communities differed for inhabitants of the canopies (ANOSIM Global P = 0.001; SIMPER 81% dissimilar) and inhabitants of trunks (ANOSIM Global P = 0.002; SIMPER 82% dissimilar) (Fig. 2). About 60% of the dissimilarity in canopy communities was due to willow canopies housing higher abundances of parasitoid wasps (Hymenoptera: pompillid, brachonid and ichneumonid), the introduced Argentine ant (*Linepithema humile*, Hymenoptera), leaf hoppers (Hemiptera, Cicidellidae: Idiocerus sp. and a type of deltocephanline hopper), a jumping plant louse (Hemiptera: Psyllidae), a minute brown scavenger beetle (Coleoptera, Latrididae), an inch worm caterpillar (Leptidoptera: Geometridae), and an array of midges (Diptera); while palm canopies hosted a bark louse (adult and nymphal Psocoptera), a hemerobiid lace wing (Neuroptera), and a crab spider (Aranae: Thomsidae) (Fig. 2).

About 60% of the dissimilarity in trunk communities was due to willow hosting higher abundances of the introduced Argentine ant, several dipterans (a dolichopodid and two midges), one leaf hopper (Hemipteran: Detocephaline hopper), a jumping plant louse (Hemipteran: Psyllidae), a caddisfly (Trichoptera), and two types of pompilid wasps; and palm trunks hosting higher abundances of the oak leaf-roller moth (*Epinotia emarginana*), a bark louse (Psocoptera), a leaf hopper (Idiocerus sp., Cicinellidae) that feeds on cottonwoods and willows, and a cobweb spider (Aranae: Theridiidae) (Fig. 2).

### Environmental Influence on Fauna

The larger area and/or denser canopies associated with palm were associated with lower total abundances of canopy fauna (Table 4A) and diversity (H’) of canopy and trunk fauna (Table 4A,B). The larger, more rugous trunk structure of the palm was associated with lower total abundances of trunk fauna and lower diversity (no. species) of canopy and trunk fauna (Table 4A,B). Canopy-community differences were mostly attributed to the greater toughness of leaves on palm than willow (BVSTEP R = 0.22), while trunk community differences were attributed to both the more rugose trunks and tougher leaves on palm than willow (BVSTEP R = 0.49).

**Table 4:** Results of forward, stepwise multiple regressions showing relationships between environmental and faunal variables associated with the canopies (A) and trunks (B) of the introduced Canary Island date palm and native arroyo willow found along the San Diego River, June 2011.

A. Canopy															
Response variable	R^2^		P		F		n		df		Independent variable(s)		+/−		
Abundance (no. ind. sample^−1^)	0.14		0.039		4.7		30		1,28		% open ground		–		
Diversity (H’)	0.25		0.005		9.3		30		1,28		canopy area		–		
Diversity (no. species sample^−1^)	0.27		0.003		10.2		30		1,28		trunk rugosity		–		
**B. Trunk**															
**Response variable**		**R^2^**		**P**		**F**		**n**		**df**		**Independent variable(s)**		**+/−**	
Abundance (no. ind. sample^−1^)		0.19		0.015		6.8		30		1,28		trunk rugosity		–	
Diversity (no. species sample^−1^)		0.29		0.002		11.3		30		1,28		trunk rugosity		–	
Diversity (H’)		0.21		0.012		7.3		30		1,28		light attenuation		–	

Note that although all environmental variables shown in Table 1 were used in the analyses, only one variable per test ended up meeting the model criteria of p≥0.05 and r^2^≥0.03.

## Discussion

### Influence of the Palm on Invertebrate Communities

The presence of the Canary Island date palm was associated with lower riparian invertebrate abundance and diversity, especially for canopy and trunk dwelling communities, and shifted the composition of these communities from a mix of beetles, midges, leaf hoppers, parasitoid wasps, and spiders to communities dominated by bark lice, the adult oak-roller moth and spiders. The palm, however, seemed to discourage inhabitation by the introduced Argentine ant despite the ant’s affinity for nesting in plant litter [28]. The greener undergrowth associated with willow may have provided the moisture required by the ant during the dry season [28]. The palm’s dense and wide canopy, tough leaves, rugous trunk, and accumulations of frond litter were all variously linked to the declines in diversity and abundance and the community shift, a finding consistent with the desert spring invasion [3]. The structure provided by palm trunks allows for detrital accumulations and, likely, epiphytes, such as fungus [20], [29], which may have attracted the bark lice [30]. More trunk rugosity may also have provided structure for hunting and web-building predators like neuropterans and spiders [22], and refuge or roosts for the adult leaf-roller moth and leafhoppers that specialize on native riparian shrubs (e.g., [31], J. Powell and R. Gill pers. comm.). Willow, on the other hand, may have provided a mix of trophic and structural resources conducive to a more diverse community than that on palm. The softer, more palatable leaves of willow (i.e, more available food), and the complex network of smooth trunks and branches [32]–[34] may have fueled this balance. Further, the sparser canopy cover of willow allowed enough sunlight through to fuel undergrowth, and greater plant species in an area may increase food types and complexity [35]. Common riparian vegetation in Southern California includes other winter deciduous woody species, such as mulefat (*Baccharis salicifolia*), sandbar willow (*Salix hindsiana*), and black willow (*Salix gooddingii*) [36], all of which have soft, palatable leaves and similar structure to arroyo willow, making it likely that they differ from the palm in similar ways as arroyo willow.

Neither the piles of accumulated, fibrous leaf litter nor any of the other characteristics of the palm seemed to affect the ground dwelling invertebrate community during this study. It may be that the ground beetles, spiders and Argentine ant, which all prefer cover and were found throughout the study area, obtained suitable cover and/or food from both the accumulated palm litter and the living grass and fine willow leaf layers beneath the willow during this time period [37]–[38]. The use of pitfalls also biases the catch toward larger-bodied, more-mobile arthropods [39]–[41], so while differences between palm and willow were not observed for these groups, smaller-bodied, less-mobile taxa (e.g., wood lice, smaller beetles) may not have been well assessed with this method. Furthermore, it is likely that some of the larger animals inhabiting this ecosystem, such as lizards and rodents, may be more affected by the differences in conditions on the ground (e.g., litter biomass and complexity, light and temperature levels, understory growth) [42]–[43] than the invertebrates.

### Ecosystem-level Effects of Palm

The presence of the palm alters the ecosystem directly and indirectly through both trophic and structural means [3], [44]. For example, the low food quality and novel structure of the palm resulted in less herbivory, and more fungivory (e.g., by bark lice), predation (e.g., spiders and lace wings), and other uses of structural resources (e.g., roosting by adult oak-roller moths and willow-eating leaf hopper) than on the willow. In total, the presence of the palm itself and the subsequent shift in the invertebrate taxa and their functions can alter physical conditions (e.g., light levels, temperature), primary production and nutrient turnover, the base of the aboveground food web (i.e., from leaves to fungus and detritus), and the pathway connecting basal and upper trophic levels (i.e., the species and trophic groups present) [3], [17], [22], [45].

These effects may extend to vertebrates within the ecosystem through direct interactions (e.g., [45]) or indirect interactions via the invertebrate fauna. Indirectly, shifts in invertebrate prey abundance and types may affect insectivorous vertebrates, such as lizards and birds. For example, the Federally endangered Least Bell’s Vireo (*Vireo bellii pusillus*), an obligate riparian species in this region, exclusively feeds on insects such as hemipterans, beetles and, in particular, caterpillars [46]–[47]. Shifts in insect composition or, more likely, decreased abundances as observed on palm may impact birds such as this one. Direct effects of palm presence on vertebrates would particularly influence those that depend upon native shrubs for nesting habitat and food capture (e.g., [48]). For example, the Least Bell’s Vireo nests about one meter above the ground in native riparian shrubs, frequently willow [49]–[50], requires dense and diverse understory to conceal the nests and foraging activities, and captures prey by picking insects off the bark or leaves and/or hovering [50]–[51]. These are trophic and non-trophic activities that could all be impeded by the impenetrable, detritus-covered, tough (downright sharp!) structure of palm canopies, trunks and litter (e.g., [50]).

### How Generalizable are Palm Effects?

Despite the different nature of our California study systems– desert springs and a coastal riparian system– the findings in this study were similar to those of [3]. Both studies found that introduced palm reduced arthropod diversity and abundance, and shifted compositions to fewer herbivores, and more predators and fungivores because of similar palm-induced changes to physical properties (e.g., more shading), and to structural and trophic resources (e.g., less canopy complexity, lower food quality). Further, [3] found that the effects of palm on arthropods did not vary much with site or season, despite variations in native plant compositions across sites and that their study site was Death Valley, one of the most annually extreme environments in the world. Given that palm effects are stronger than the temporal and regional spatial variations examined across these studies, it is likely that similar effects of palm on invertebrates will be observed in other ecosystems as well. Predictions of susceptible ecosystems and regions are therefore useful.

As seen in California, moist ecosystems within arid and semi-arid climates may be susceptible to palm invasion and similar effects on the physical environment and invertebrate community [3] (this study). Many ecosystems within tropical and subtropical climates are suitable for introduced palms because of the combination of warm temperatures and moisture (e.g., [12], [52]). Within these regions, palms may have the most dramatic effects where native vegetation is most different. For example, areas dominated by palatable mid-story and/or ground cover plants, such as grassland, scrub [3], (this study), or broadleaf tropical forest [52]. Further, effects of invasion may be most dramatic in “island” ecosystems, where there are relatively small patches of ecosystem surrounded by a highly contrasting matrix. These “islands” are vulnerable to invasion and degradation (due to edge effects), and to native species declines (due to stochasticity), and lack of recolonization because of their isolated nature (e.g., [53]–[54]). Island ecosystems susceptible to palm invasion may be true islands [45], desert springs surrounded by an arid landscape [55]–[56], or almost any fragmented or encroached ecosystem that receives moisture from stream, groundwater or tidal flows (e.g., inland and coastal wetlands, canyons bottoms, and fragmented riparian areas) (e.g., [2], (this study)). In particular, urban ecosystems are at risk of invasion because of the combination of high propagule pressure and artificial moistening of an otherwise dry landscape through irrigation and runoff. Further, controlled river flows and urban runoff in populated areas have shifted many arid- and semi-arid rivers from historic ephemeral flows to perennial low flows [57]. This shift reduces the frequency and intensity of flood and scour events that would clear out invasives, and creates the moist environments favored by many introduced plants [57], such as palms.

### Future Directions and Recommendations

Further testing of the effects of palm presence on the invertebrate community during other times of the year, and the effects of both palm presence and its associated invertebrates on common and threatened riparian vertebrates, and on other ecosystem processes (e.g., decomposition rates, nutrient cycling), are needed to assess the threat level of these relatively understudied invaders. Understanding the effects of the palm at this point, while it mostly exists in localized patches, is important for making management decisions. A species does not have to be abundant to have a relatively large impact on its ecosystem (e.g., ecosystem engineers, [58]) and, if this is the case with the palm, now would be the time to initiate removal and control strategies [59]–[60]. The California Coastal Commission currently prohibits the planting of [2] listed plants in new coastal developments, but existing developments are plentiful and may need to be addressed. Control recommendations include source removal efforts, or propagule removal efforts if removal is too expensive for some landowners and land managers. However, total removal would be ideal to avoid the current effects of living palms and “ghost palm” effects, where dead palm material persists for many years reducing habitat quality [3], [11]. Another control approach would be to discourage the further planting of this species in developments adjacent to riparian corridors or other moist ecosystems where short-distance dispersal of the abundant propagules is sure to occur. This sort of effort would require a coordinated outreach effort among the nurseries offering this species, the public, ecologists and environmental managers [61].

Study of the rates and patterns of spread of the palm are also needed. Anecdotal data from Southern California reveals mostly localized spread of the palm around parent plants [2], but with somewhat recent expansion of the distribution, in particular into the estuarine reach of the river [15]. Information on spread rate and patterns, coupled with information from this study on the local effects, could help to predict larger-scale effects of spread. Although the palm is not currently one of the most aggressive invaders in the waterway, it has a prolific seed set and is displaying salinity tolerance, two traits that could contribute to successful spread in this coastal river system (e.g., tamarisk; [17]). Many species start in a lag phase and unforeseen conditions trigger rapid spread [60]. More thoroughly understanding the effects of palm invasion will be important for prioritizing management decisions.
